# Effect of mechanical vibration on peripheral blood flow in laboratory exposure: a narrative review

**DOI:** 10.13075/ijomeh.1896.02702

**Published:** 2026

**Authors:** Fawaz O. Alenazy, Abozer Y. Elderdery, Arun Vijay Subbarayalu, Badr Alzahrani, Emad Manni, Jeremy Mills, Mariam A. Ameer, Shereen M. Said

**Affiliations:** 1 Jouf University, Department of Clinical Laboratory Sciences, College of Applied Medical Sciences, Sakaka, Saudi Arabia; 2 Arabian Gulf University, College of Education, Administrative and Technical Sciences, Center for Quality Assurance and Strategic Planning, Manama, Kingdom of Bahrain; 3 University of Portsmouth, School of Medicine, Pharmacy and Biomedical Sciences, Portsmouth, United Kingdom; 4 Jouf University, Department of Physical Therapy and Health Rehabilitation, College of Applied Medical Sciences, Sakaka, Saudi Arabia; 5 Misr University for Science and Technology, Department of Basic Science, Faculty of Physical Therapy, Giza, Egypt

**Keywords:** whole-body vibration, mechanical vibration, local vibration, peripheral blood flow, peripheral circulation, blood circulation

## Abstract

Although mechanical vibration (MV) has grown in popularity and utilization in recent years, there is lack off the literature in relation to its impact on peripheral blood flow. The main aim of the current narrative review is to examine the effects of local mechanical vibration (LMV) and whole-body vibration (WBV) on peripheral blood flow in different populations. Between January 2000 – February 2025, the authors used the following keywords related to MV and peripheral blood flow: “whole-body vibration,” “WBV,” “whole-body periodic acceleration,” “WBPA,” “local vibration,” “LV”, “mechanical vibration,” “MV,” “blood flow,” “peripheral blood flow,” and “peripheral circulation” to search Google Scholar, Web of Science, Scopus, and PubMed databases and reference lists from relevant articles. The authors used both single-word and combination searches. Nineteen potential articles that fit the inclusion criteria were identified. While LMV exhibited positive as well as negative impacts on peripheral blood flow in several studies, WBV was demonstrated to have a favorable effect on peripheral blood flow. Furthermore, the impact of MV on blood flow was modified by variables such as vibration type and frequency. Mechanical vibration has benefits and risks to the peripheral blood flow. When it comes to enhancing peripheral blood flow, WBV is more beneficial than LMV. Standardized procedures and uniform results reporting can be more advantageous in order to facilitate future meta-analyses and allow for more transparent comparisons between studies.

## Highlights

- There is a lack of literature on the effects of mechanical vibration (MV) on peripheral blood flow.
- Whole-body vibration generally shows a positive effect on peripheral blood flow.
- Local MV has both positive and negative impacts on blood flow.
- Vibration type and frequency significantly influence peripheral circulation.
- Further studies are needed to delineate the effects of MV on peripheral blood flow.

## INTRODUCTION

Mechanical vibration (MV) is described as a mechanical oscillation, or a periodic change in force, acceleration and displacement over time [1]. Local mechanical vibration is a forced oscillation in which energy is transferred from an actuator, the vibration device, to a resonator, and certain sections of human body [1,2]. Amplitude, frequency and phase angle are used to characterize the sinusoidal shape of the oscillations found in the majority of vibration devices. The mechanical wave represented by the mathematical amplitude, or half of the peak-to-peak amplitude. The human body is accelerated during vibration, which results in a reactive force generated both inside and outside the body [3]. Blood flow in the heart, lungs, and skeleton increases as exercise intensity rises. Skeletal and cardiac muscle get 85–90% of the entire cardiac output at peak exercise intensities to meet extreme metabolic demands by increasing oxygen and nutrient delivery through selective vasodilation in working muscles and simultaneous vasoconstriction in inactive tissues [4].

The physiological reaction of humans to MV has long captured the interest of researchers, particularly in light of its potential as a non-pharmacological strategy to increase peripheral blood flow [5]. In this context, MV has been reported to support the function of different vital organs. For example, Figueroa et al. [6] reported that MV significantly reduces both blood pressure and arterial stiffness among obese females and overweight young individuals. Additionally, a study confirmed the efficacy and practicality of MV as a potential alternative exercise modality for managing nonalcoholic fatty liver disease [7]. Use of MV also enhanced markers of mitochondria, lowered liver fat and decreased the levels of glucose/insulin in diabetic mice [8].

On the other hand, occupational health studies have shown that workers’ health and well-being can be negatively impacted by prolonged MV when paired with other elements like awkward postural demands [9]. For instance, high-magnitude vibration can cause long-term harm to the upper body muscle groups, joints, and peripheral circulation in a common disorder known as hand-arm vibration syndrome (HAVS) [10]. Employees with HAVS may suffer from cold-induced vasospasms and hand and finger blanching [11]. Helmkamp et al. [12] recognized that the specifics of the vibration exposure have a significant impact on the health effects of occupational vibration (e.g., vibration amplitude, direction, and frequency). Indeed, there may be several advantages to brief exposure to low magnitude MV, especially in terms of improving local muscle blood flow [13].

Because these measurements shed light on the metabolic alterations taking place in skeletal muscle, the impact of MV on peripheral blood flow and muscle oxygen consumption is particularly interesting and has lack of literatures [14]. Muscle oxygenation and blood flow are intimately associated [14–16]. Increased needs for oxygen and fuel, as well as higher concentrations of carbon dioxide and hydrogen ions, among other reasons, cause blood flow to the working muscle to increase during exercise [17]. A potential mechanism of action for MV treatment may be indicated by changes in peripheral blood flow brought on by MV administration [14]. Increasing circulation at the site of musculoskeletal injury throughout the fibroblastic repair and maturation-remodeling phases of recovery is a common clinical objective of using treatment methods [18]. When increasing blood flow or muscle oxygenation is the clinical objective, MV may be utilized as a therapeutic intervention [14].

Low-frequency vibrations (60 Hz) have an impact on human peripheral blood circulation [19]. Those subjected to MV showed an increase in diastolic pressure and a growth in blood flow in the vascular system [20]. Peripheral blood circulation is accelerated by rhythmic muscle contractions. According to studies, 9 min of low-frequency (26 Hz) vibrations of the entire body enhance blood volume and flow in the lower extremity muscles [21]. A relatively recent development in the treatment and rehabilitation of musculoskeletal ailments is MV therapy [22].

Nevertheless, little is known about how it works on peripheral circulation. Clinical professionals may find it challenging to decide whether to incorporate MV into their practices due to inconsistent research findings regarding whether MV affects muscle oxygenation and peripheral blood flow [14]. Additionally, there is a lack of discussion regarding the differences between whole-body vibration (WBV) and local mechanical vibration (LMV) in terms of enhancing peripheral blood flow, making it difficult to determine which type of MV is more advantageous for doing so. To help clinicians comprehend this potentially significant therapy technique and identify research gaps, a narrative review of the literature is required to summarize the body of literature. Thus, the authors’ study's goal was to evaluate the body of research on the effects of MV (LMV and WBV) on peripheral blood flow in different health conditions such as healthy subjects, diabetic, spinal cord injury (SCI) patients, and overweight/obese subjects. In clinical practice and occupational health, a deeper comprehension of the physiological effects of MV on blood flow may lead to better patient outcomes and may change the negative concepts regarding MV at workplace. Therefore, the novelty of the current study is that it summarizes, in a single narrative review, the actual effects of both WBV and LMV on peripheral blood flow.

## METHODS

### Literature search and searching strategies

**Figure 1.**
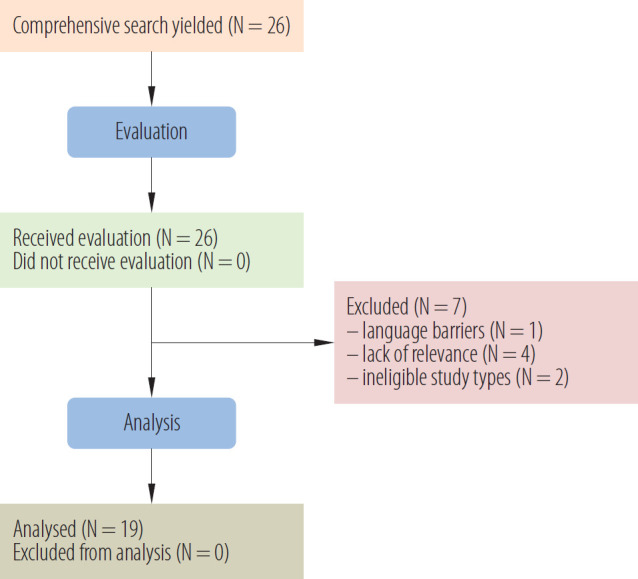
Flowchart of selected articles on mechanical vibration (local and whole-body vibration) that assess the peripheral blood flow, review 2000–2025

The databases Google Scholar, Web of Science, PUBMED, and SCOPUS were used to review the literature on the MV (LMV and WBV) effect on the peripheral blood flow of humans or peripheral circulation. The authors looked for the following keywords in each database “MV” or “vibration” and “peripheral blood flow” or “peripheral circulation” as well as one of the 3 methods to induce non-invasive stimulation to the peripheral blood flow: “LV”, “local vibration,” “WBV,” “whole-body vibration,” and “whole-body periodic acceleration,” “WBPA.” The authors reviewed the abstracts and titles to find pertinent details on the different pulsatile shear stress therapies in connection to peripheral circulation. A comprehensive search yielded a total of 26 articles. Nineteen articles in all were included in this narrative review as shown in Figure 1.

### Inclusion and exclusion criteria

This narrative review comprised experimental and review studies that met the following criteria:

– inclusion criteria
  - the full-text articles which included a detailed description of the different parameters of the MV training (LMV and WMV),
  - articles and reviews released from January 2000 to February 2025,
  - articles which included different health conditions such as normal subjects, diabetics, SCI, obese or overweight subjects,
  - research articles written in English,
– exclusion criteria
  - lack of relevance,
  - inadequate attention to local and WBV,
  - lack of direct assessment of the impact of MV on peripheral blood flow,
  - articles not written by English,
  - articles that made use of duplicate sample data,
  - Editor's letters or recommendations or conference abstract.

## RESULTS

### Local vibration stimulation effect on peripheral blood circulation

Local mechanical vibration straddles the line between benefit and harm due to its effect on the peripheral circulation, as it is evident that perfusion is required to meet the higher demands of vibration exercise, which mandates muscular energy turnover and heat production. Therefore, elucidating the beneficial and detrimental effects of LMV on vascular tissue and blood flow was the primary goal of the current literature.

Vibration of 16–250 Hz has been believed to impede perfusion since acute hand-transmitted vibration has been demonstrated to decrease digital blood flow, most likely due to vasoconstriction [23]. In addition, Luo et al. [24] showed that increased vibration intensity tended to accelerate the reduction in finger blood flow in both hands, and repeated vibration exposure had cumulative effects on the unexposed hand's reduction in finger blood flow. This may be due to the increase of sympathetic activity which induced vasoconstriction in the blood vessels of the hands and feet [25,26]. Ye and Griffin [27] further explained these results which suggest that the Pacinian corpuscle transmits vibrotactile sensations near threshold at higher frequencies (63 Hz, 125 Hz, and 250 Hz), leading to vasoconstriction, but the Pacinian corpuscle does not mediate feelings near threshold at lower frequencies (8 Hz, 16 Hz, and 31.5 Hz) [27]. Some studies concluded that when the fingers are exposed to vibration, vasoconstriction of blood vessels is influenced by both how strong the vibration is and its frequency. Finger blood flow was reduced by vibration at magnitudes lower than those on many powered hand tools [28–30]. Additionally, exposing just one hand to vibration caused similar blood flow reductions in both the exposed and non-exposed hands, indicating that the body's central nervous system likely plays a role in this reaction. After the vibration stops, the restricted blood flow persists, and the extent of this ongoing vasoconstriction is still related to the strength and frequency of the original vibration exposure [30].

In contrast, study conducted by Espeit and Lapole [31] showed that rise in popliteal venous blood velocity following the use of local vibration and progressive compression stockings. The strongest impacts were produced by their combination due to an increase in local metabolism (i.e., reflex muscular contractions caused by vibrations) and/or vasodilatation of the microcirculation (i.e., nitric oxide [NO] secretion reported following exposure to mechanical stress and neuropeptide release induced by a local axon reflex through activation of polymodal receptors) [31], vibration has been proposed to increase arterial and microcirculatory blood flow. Also, Needs et al. [32] showed that 38 Hz and 47 Hz localized vibrations greatly raise blood flow in the popliteal artery without changing heart rate and may aid in muscle recovery. According to Button et al. [10] a hand-held vibration device placed beneath the right foot or gluteal muscle improved local blood flow, which could be helpful in the treatment of pain or other symptoms brought on by acute or chronic musculoskeletal injuries. On the other hand, Fateh et al. [33] demonstrated no significant change in the peripheral blood flow for diabetic participants through the application of local calf vibration although it tended to increase the nerve conductivity and balance. Ren et al. [8] showed improved skin blood flow (SBF) in the feet of diabetic patients after the application of local intermittent MV. Vibration-induced vessel vasodilation and the ensuing increases in SBF are primarily controlled by 2 mechanisms:

– pulsating mechanical forces act on endothelial cells (ECs) to release NO and NO synthase (NOS), which contributes to vessel vasodilation;
– vibration-induced simulation of polymodal receptors on the skin surface may result in the release of neuropeptides, which in turn may induce microvascular vasodilation related to nerve axon reflex [34].

In addition, Liao et al. [35] showed that structural regularity of SBF at the first metatarsal head of healthy people was considerably enhanced by a local vibration at 100 Hz. This could be related to the plantar skin's mechanoreceptors. Many Pacinian corpuscles, which are more sensitive to higher vibration frequencies, are found in the skin, as a consequence, it might be simpler to elicit an SBF response at a greater vibration frequency [35]. Furthermore, a higher vibration frequency might trigger a stronger SBF response by activating more mechanoreceptors, such as Pacinian and Messinian corpuscles [35].

The majority of previously mentioned research [23,27,30] that has shown a decrease in peripheral blood flow as summarized in Table 1 employed high vibration frequencies, ranging 250–315 Hz [36]. Vibratory microangiopathy develops when the hand is exposed to high vibration stimuli for an extended period of time, causing peripheral microcirculatory and neurological dysfunction [37]. It is clinically significant to examine how local vibration stimulation affects the hands and other body parts peripheral blood microcirculation. Local vibration may disrupt the activity of neurons, skin receptors, and nerve trunks, which causes synaptic nerve terminals to secrete more norepinephrine [38]. Norepinephrine enters the bloodstream in large quantities due to the incapacity of synaptic nerve endings to effectively collect it which results in elevated vascular tension, causing vasospasm and decreases peripheral blood flow [39]. Additionally, ECs are susceptible to developing an inflammation following local vibratory stimuli, and one of the main components of the inflammatory response is inflammatory factors [40].

**Table 1.** Overview of mechanical vibration (local and whole-body vibration) studies (N = 19) that assess the peripheral blood flow, review 2000–2025

Study	Age [years]	Participants [n]	Participants’ health condition	Sex	Vibration type	Frequency [Hz]	Amplitude [mm]	Duration of exposure	Weights	Blood flow outcome
Bovenzi et al. [23]	31.5	10	healthy	males	local	16–250	not mentioned	15 min	yes	reduced
Luo et al. [24]	30.7	10	healthy	6 males, 4 females	local	60	not mentioned	15 min	no	reduced
Ye and Griffin [27]	18–30	15	healthy	males	local	8, 16, 31.5, 63, 125, 250	not mentioned	3 min	no	reduced
Thompson and Griffin [30]	26–32	12	healthy	males	local	16, 31.5, 63, 125, 250, 315	not mentioned	30 min	no	reduced
Button et al. [10]	40–65	20	healthy	10 males, 10 females	local	60	not mentioned	30 min	no	improved
Needs et al. [32]	22.3	26	healthy	14 males, 12 females	local	30, 38, 47	not mentioned	5–10 min	no	improved
Espeit and Lapole [31]	22.5±2.2	24	healthy	17 males, 7 females	local	100	1	15 min	yes	improved
Ren et al. [34]	55–75	30	15 healthy, 11 diabetic	13 males, 13 females	local	50	2	5 min	no	improved
Liao et al. [35]	18–35	12	healthy	n.d.	local	100	1	10 min	no	improved
Fateh et al. [33]	60.3	17	diabetic	11 males, 6 females	local	60	not mentioned	15 min	no	not changed
Kerschan-Schindl et al. [47]	25–35	20	healthy	12 males, 8 females	whole body	26	3	9 min	yes (body weight)	improved
Mahbub et al. [52]	≥65	30	healthy	15 males, 15 females	whole body	15, 20, 25	4	5 min	yes (body weight)	improved
Lohman et al. [21]	23.93	45	healthy	23 males, 22 females	whole body	30	5–6	10 min	yes (body weight)	improved
Lythgo et al. [54]	21.8	9	healthy	males	whole body	5–30	2.5–4.5	12 min	yes (body weight)	improved
Johnson et al. [53]	71	10	diabetic	3 males, 7 females	whole body	26	2	10–30 s	yes (body weight)	improved
Manimmanakorn et al. [55]	63.2	36	diabetic	13 males, 23 females	whole body	30–40	2–4	12 min	yes (body weight)	improved
Sañudo et al. [57]	67–72	40	diabetic	21 males, 19 females	whole body	12–16	4	12–20 min	yes (body weight)	improved
Herrero et al. [58]	36.1	8	spinal cord injury	6 males, 2 females	whole body	10, 20, 30	5	3 min	yes (body weight)	improved
Figueroa et al. [6]	18–35	10	overweight/obese	females	whole body	25–30	1–2	30 s – 1 min	yes (body weight)	improved

Conversely, tissue microcirculation is positively impacted by low-frequency, low-amplitude local vibration which encourages the restoration of blood microcirculatory system function in addition to stimulating tissue metabolism [41] as shown in the previously mentioned research [10,31–35] that summarized in Table 1. It has been demonstrated that certain levels of vibration stimulation aid in the healing of diabetic wounds [42]. Ichioka et al. [43] and other study [44] reported that vibration stimulation enhances blood microcirculatory activity and that NO is a key player in the vibration-induced vasodilatation process. Clarifying how vibration stimulation affects peripheral circulation blood flow promotion or inhibition, minimizing harmful vibration stimulation, and enhancing the body's beneficial vibration stimulation will therefore be crucial for the prevention of vibration diseases and improving the patient clinical outcomes.

### Whole vibration stimulation effect on peripheral blood circulation

Whole-body vibration can be carried out by having participants stand on a plate or platform that revolves around a central fulcrum, producing vertical motion on both sides of the fulcrum, or that produces sinusoidal oscillations in a vertical up-and-down motion [45]. Vibrations from these motions are indirectly transferred to the subject's entire body different tissue through the legs [46]. A combined physical activity with vibration training seems to decrease the imposed vibration and stance misalignment when standing on a vibrating platform. On the other hand, standing on a vibrating platform may cause rhythmic muscle contractions that could improve peripheral blood flow and compensate for the lack of other physical activity [47,48]. Several studies were investigated in this review to explore the effect of WBV on the peripheral blood flow, and used as a reference to summarize the appropriate parameters for detecting the benefits and harmful effects of the MV on the human circulation as shown in Table 1.

A study conducted by Kerschan-Schindl et al. [47] that used WBV to detect its effect on the peripheral blood flow in healthy subjects found that standing on a vibrating platform for a few minutes causes the relative moving blood volume of the gastrocnemius and quadriceps muscles to increase. In the popliteal artery, the resistance index dropped and the mean blood flow rose as well. The most plausible explanation could be that the popliteal arter's mean speed of flow may rise as a result of the peripheral resistance being decreased by the muscles’ small arteries enlarging [47]. Furthermore, thixotropism might potentially be involved during WBV training in which vibration stress or agitation tends to decrease the blood viscosity. Vibration may enhance the mean blood flow rate in the popliteal artery by decreasing blood viscosity [49]. The decrease in the resistive index of the popliteal artery is most likely also due to the decrease in peripheral resistance.

Certain skin mechanoreceptors are stimulated when exposed to various WBV frequencies, with the most prevalent mechanoreceptors in glabrous skin, the Meissner corpuscles [50]. With a maximal sensitivity of about 30–40 Hz, these mechanoreceptors are preferentially responsive to low-frequency vibrations of 5–40 Hz [51]. Vibration stimulation-induced exchanges of neuronal signals, hormones, and mediators involving both local and systemic vasoregulatory processes are the origin of such improvements in SBF [52–54]. A study conducted by Mahbub et al. [52], found that WBV at 20 Hz can effectively increase peripheral blood flow if its magnitude is within the advised range. Also, one of the potential explanations is that the MV forces acting on the ECs are caused by cellular friction, which raises NO levels noticeably. A significant rise in circulating NO concentrations that causes the dilatation of resistant blood arteries could be the cause of this cardiovascular alteration [21].

Johnson et al. [53] conducted a study using WBV on diabetic subjects, and found that SBF on the foot's dorsum was noticeably higher at the beginning, but by the fifth minute after exposure, it had dropped back to baseline. The authors believed this return is caused by modifications in centrally controlled SBF because there is evidence that vibration frequency affects the sympathetic nervous system's activation [53]. Additionally, there was evidence that WBV reduces neuropathic pain; however, it is unclear how higher SBF and decreased neuropathic pain are related [44]. Increased SBF may be linked to less pain, and NO-induced vascular dilatation may be the cause of that increased SBF [45]. Moreover, Manimmanakorn et al. [55], confirmed the effect of WBV in improving the systolic and diastolic resting blood pressure through dilating small blood vessels inside the muscles and thus decreasing the peripheral vascular resistance. According to the suggested underlying mechanism, acute vibration exposure may lower peripheral vascular resistance by preventing smooth muscle release of vasoconstrictor material (endothelia) and boosting arteriole vasodilation through the release of prostaglandins and endothelial NO [56]. Furthermore, Sañudo et al. [57] confirmed the improvement of peripheral blood flow in diabetic subjects using the WBV training for the same previous causes in addition by decreasing the visceral fate that associated with increasing the arterial stiffness.

**Figure 2.**
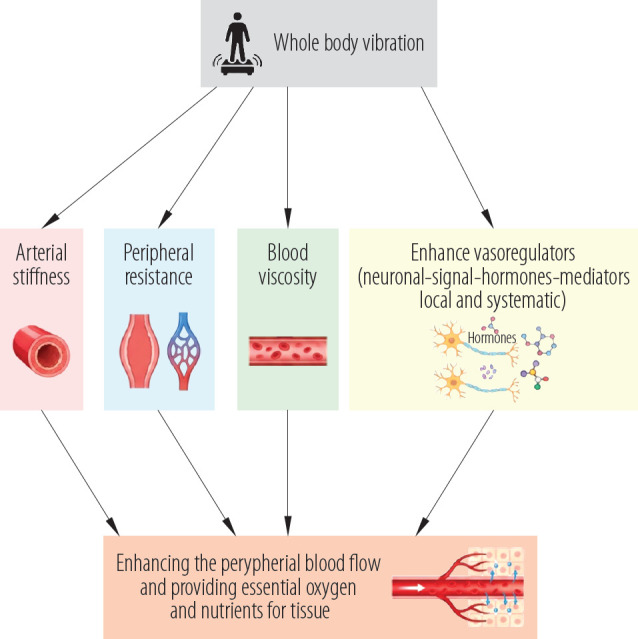
Effect of whole-body vibration on peripheral blood flow

While studying the effect of WBV on other different cases, Herrero et al. [58] suggested that WBV is a useful technique for stimulating the growth of muscles and increasing leg blood flow in SCI patients, and it may be included in their rehabilitation regimens. In addition, Figueroa et al. [6] demonstrated that in young overweight/obese normotensive women, 6 weeks of WBV training improved wave reflection and sympathovagal balance, which in turn reduced systemic arterial stiffness and improving the peripheral blood flow. Whole-body vibrations training may improve muscle strength and artery function in deconditioned people unable to engage in traditional exercise. Figure 2 summarized the positive effect of WBV in enhancing the peripheral blood flow and providing essential oxygen and nutrients for tissue healing and overall health.

Graphic source: BioRender.

All the studies considered investigating the impact of WBV showed an improvement in peripheral blood flow, demonstrating that WBV is more efficient at enhancing circulation without posing any risks than LMV applications. There appears to be no study that compares the effect of LMV and WBV effect on the peripheral blood flow. However, this review suggests that the positive impact of WBV is greater than that of LMV, specially the WBV frequency 5–40 Hz, and resulted in mean therapeutic effect of WBV on peripheral blood flow. This result agrees with a meta-analysis was conducted by Games et al. [14] showing that lower frequencies result in more peripheral blood flow than higher frequencies. This data implies that in order to improve peripheral blood flow, lower frequencies ought to be employed. One theory is that the rate at which muscles contract could have an impact on the increased blood flow. Greater perfusion may be possible with lower frequencies since they may give longer intervals between contractions. However, this perfusion might not be possible at higher frequencies, which would lead to reduced blood flow when WBV is applied. Blood velocity in the femoral artery was studied by Lythgo et al. [54] in relation to vibration frequency and amplitude where they identified that lower frequencies (10–30 Hz) enhanced blood velocity more than higher frequencies (20–30 Hz).

The participants’ positions and whether they are loaded or unloaded with body weight are significant WBV variables. The authors’ further sub-analyses demonstrated that loading affected the responses of peripheral blood flow as shown in the meta-analysis by Games et al. [14] and this may lead to the stimulation of more muscle contraction during the weight bearing that enhances the peripheral blood flow and damping the effect of the vibration inside the body. Additionally, a review of the literature on exercise-induced hyperemia revealed that muscular contractions may release a variety of possible vasodilator substances, such as potassium [59], adenosine [60], and NO [61]. The frequency of muscular contractions may not be equivalent to the frequencies that occur during a session of WBV, even if exercise-hyperemia studies support increased blood flow with muscle contractions. It is clear that further research is required in this field, particularly to look into the role of WBV. Numerous processes, including metabolic, humeral, and neural variables, may contribute to the elevated blood flow seen following WBV exposure. The mechanism by which blood flow increases with therapeutic WBV exposure requires further investigation.

To determine if WBV is safe and effective in patients with disease, illness, or injury, more research is clearly necessary. The majority of the studies assessed were in healthy individuals except a few studies focused on the effect of WBV in improving the peripheral blood flow in diabetic, SCI patients, and overweight/obese subjects which indicated the positive effect of WBV in improving the peripheral blood flow and muscle strength in deconditioned subjects but still further research studies are needed. When interpreting these findings, it is important to take consider the limitations of the study and to use caution when extending the findings as the research considered used a wide range of vibration settings and measurement instruments. Finally, there is the impact of bias at both the study level and in the analysis as not all studies may have been retrieved for inclusion such as publications in journals not indexed by search engines and articles not published in English.

From all previous studies it is not definitive that WBV is better than LV for improving blood flow, as both can increase circulation, but the positive and negative effectiveness depends on the type of vibration, frequency, duration, and the specific area of the body being targeted. Both methods can stimulate blood flow by inducing rhythmic muscle contractions and creating shear stress on blood vessels, but different vibration parameters yield different results in both WBV and localized vibration. Peripheral circulation may be adversely affected by high frequency LMV (250–315 Hz), especially in the hands and other peripheral body regions with tiny blood capillaries. Further studies are indeed warranted to rigorously confirm and delineate the effects of MV on peripheral blood flow, utilizing a range of parameters to ensure robust and generalizable results.

## CONCLUSIONS

Mechanical vibrations includes both LMV and WBV influences peripheral blood flow in complex ways. Generally, while WBV demonstrates favorable outcomes, the effects of LMV remain inconsistent with both beneficial and adverse responses reported. These variations are mostly influenced by factors such as vibration type, frequency and population characteristics. As such, careful consideration of these parameters is crucial when applying MV for therapeutic or performance purposes. Upcoming research must aim to standardize protocols and more explore the mechanisms behind the impact of MV on vascular responses.
